# Prevalence of *BRCA1/BRCA2* mutations in a Brazilian population sample at-risk for hereditary breast cancer and characterization of its genetic ancestry

**DOI:** 10.18632/oncotarget.12610

**Published:** 2016-10-12

**Authors:** Gabriela C. Fernandes, Rodrigo A.D. Michelli, Henrique C.R. Galvão, André E. Paula, Rui Pereira, Carlos E. Andrade, Paula S. Felicio, Cristiano P. Souza, Deise R.P. Mendes, Sahlua Volc, Gustavo N. Berardinelli, Rebeca S. Grasel, Cristina S. Sabato, Danilo V. Viana, José Carlos Machado, José Luis Costa, Edmundo C. Mauad, Cristovam Scapulatempo-Neto, Banu Arun, Rui M. Reis, Edenir I. Palmero

**Affiliations:** ^1^ Center of Molecular Diagnosis, Barretos Cancer Hospital, Barretos, São Paulo, Brazil; ^2^ Oncogenetics Department, Barretos Cancer Hospital, Barretos, São Paulo, Brazil; ^3^ Institute of Research and Innovation in Health, University of Porto, Porto, Portugal; ^4^ Institute of Molecular Pathology and Immunology at the University of Porto (IPATIMUP), Porto, Portugal; ^5^ Molecular Oncology Research Center, Barretos Cancer Hospital, Barretos, São Paulo, Brazil; ^6^ Prevention Department, Barretos Cancer Hospital, Barretos, São Paulo, Brazil; ^7^ Pathology Department, Barretos Cancer Hospital, Barretos, São Paulo, Brazil; ^8^ MD Anderson Cancer Center, Houston, Texas, USA; ^9^ Life and Health Sciences Research Institute (ICVS), Health Sciences School, University of Minho, Braga, Portugal; ^10^ ICVS/3B's-PT Government Associate Laboratory, Braga/Guimarães, Portugal; ^11^ Barretos School of Health Sciences, Dr. Paulo Prata–FACISB, São Paulo, Brazil

**Keywords:** hereditary breast cancer, BRCA1/BRCA2 mutation profile in Brazil, genetic ancestry, HBOC in brazil, c.5266dupC prevalence in brazil

## Abstract

**Background:**

There are very few data about the mutational profile of families at-risk for hereditary breast and ovarian cancer (HBOC) from Latin America (LA) and especially from Brazil, the largest and most populated country in LA.

**Results:**

Of the 349 probands analyzed, 21.5% were *BRCA1/BRCA2* mutated, 65.3% at *BRCA1* and 34.7% at *BRCA2* gene. The mutation c.5266dupC (former 5382insC) was the most frequent alteration, representing 36.7% of the *BRCA1* mutations and 24.0% of all mutations identified. Together with the *BRCA1* c.3331_3334delCAAG mutation, these mutations constitutes about 35% of the identified mutations and more than 50% of the *BRCA1* pathogenic mutations. Interestingly, six new mutations were identified. Additionally, 39 out of the 44 pathogenic mutations identified were not previously reported in the Brazilian population. Besides, 36 different variants of unknown significance (VUS) were identified. Regarding ancestry, average ancestry proportions were 70.6% European, 14.5% African, 8.0% Native American and 6.8% East Asian.

**Materials and methods:**

This study characterized 349 Brazilian families at-risk for HBOC regarding their germline *BRCA1/BRCA2* status and genetic ancestry.

**Conclusions:**

This is the largest report of *BRCA1/BRCA2* assessment in an at-risk HBOC Brazilian population. We identified 21.5% of patients harboring *BRCA1/BRCA2* mutations and characterized the genetic ancestry of a sample group at-risk for hereditary breast cancer showing once again how admixed is the Brazilian population. No association was found between genetic ancestry and mutational status. The knowledge of the mutational profile in a population can contribute to the definition of more cost-effective strategies for the identification of HBOC families.

## INTRODUCTION

Breast cancer (BC) constitutes the leading cause of cancer mortality among Brazilian women. According to the Brazilian National Cancer Institute (INCA), the number of new BC cases expected in 2014 was 57,120. A higher incidence occurs in Southeastern Brazil, with an estimated risk of 71 new cases/year per 100,000 women [1].

It is estimated that 5 to 10% of the BC cases are hereditary [2, 3]. Therefore, in Brazil, approximately 6,000 new cases of hereditary BC are expected yearly, which is alarming both for its numerical proportions, and for the fact that most of these tumors are not recognized as from an hereditary origin [1, 2].

In Brazil, there are few services specialized in identifying and monitoring families at-risk for hereditary cancer. These services are mainly concentrated in capital cities of some Brazilian states, limiting or at least hindering the access of a great share of the population living in more remote areas [4–6]. Thus, data on BC familial aggregates are still scarce in Brazil. The main studies published so far involve i) specific populations, such as young women with BC [7]; or ii) regions and/or specific gene mutations, with most studies focusing their analysis on the founder mutations at *BRCA1/BRCA2* genes [7–13].

### Genetic ancestry

The ethnic substructure of the population is an important factor that can influence the incidence, prognosis and mortality of BC. A large body of evidence suggested an inverse correlation between low incidence of BC and high rates of mortality among African-American women when compared with Caucasian, with the risk of death from BC being 67% higher among African-Americans [14–16]. In addition, work developed in Mexico by Fejerman and collaborators [17], showed that for every 25% increase in European ancestry there was a 20% increase in risk of BC. Otherwise, results of a case-control study published by Bonilla and collaborators [18] involving 328 Uruguayan women showed no evidence that overall genetic ancestry differs between BC patients and controls in Uruguay.

According to Kehdy and collaborators, Brazil is a classical model for population genetics studies on admixture, since it received several immigration waves from diverse European origins during the last five centuries. In addition, African slaves arrived to Brazil during four centuries, and the geographic origin of Brazilian slaves differ from Caribbean and African American [19, 20]. However, little is known about the genetic ancestry profile in a highly admixed population with BC and/or ovarian cancer (OC). In addition, to the best of our knowledge, there are no studies characterizing the ancestry profile of a cohort at-risk for hereditary BC in the Brazilian population.

Therefore, we characterized a cohort of 349 families at-risk for hereditary breast and ovary cancer (HBOC) from a single institution for the presence of germline *BRCA1* and *BRCA2* mutations, as well as for ethnic composition (genetic ancestry) and correlated these molecular findings with the clinical and familial history of patients.

## RESULTS

### General characterization

Among the 349 index cases analyzed (following criteria described in the “Material and Methods” section), 97.4% (*n* = 340) were female and 2.6% (*n* = 9) were male, all with breast and/or OC history. The vast majority of probands had BC as the primary tumor (*n* = 292, 83.7%), 50 (14.3%) had a history of OC and 7 patients (2.0%) had other tumors than BC or OC as primary tumor site (gall bladder (*n* = 2), colorectal (*n* = 2), melanoma (*n* = 1), prostate (*n* = 1) and endometrium (*n* = 1)).

The mean age at diagnosis was 41 years (SD = 12, CI: 16–86 years). When categorized, 22.9% (80 patients) received a cancer diagnosis at an age below 30 years, 32.1% (*n* = 112) between 30 and 40 years, 24.9% (*n* = 87) were diagnosed at ages between 40 and 50 years old and 20.1% (70 patients) of the probands had their primary tumors detected after 50 years old (Supplementary Table S1).

The majority of BC types were invasive ductal carcinoma (85.2%), followed by intraductal carcinoma (7.6%), papillary (3.4%), lobular (3.4%) and medular (1.1%) carcinomas. As for OC types, most (74%) were serous adenocarcinomas, followed by undifferentiated tumors (13%), endometrioid tumors (7%), clear cell tumors (2%), mucinous tumors (2%) and peritoneal tumors (2%).

Among the 349 probands, 21.5% were germline mutated (*n* = 75) with 65.3% of them carrying mutations in *BRCA1* and 34.7% in the *BRCA2*. When segregating mutated patients according to primary tumor site, 20.5% of patients with BC were mutated (60 cases) being 63.3% of the mutations in the *BRCA1* gene and 36.7% in *BRCA2*. Among patients whose primary tumor was OC, 30.0% (15 patients) presented pathogenic mutations in the *BRCA1/BRCA2* genes, with the majority (73.3%) being located in *BRCA1*.

Taking into consideration only mutated patients with BC, among those with *BRCA1* mutated, 72.7% of the tumors were triple negative and 27.3% Luminal A or B1. Regarding the *BRCA2* mutated patients, 9.1% were triple negative, 63.6% Luminal A or B1 and 27.3% Luminal B2. For those *BRCA1/BRCA2* WT, the majority (52.6%) were Luminal A/B1, followed by Luminal B2 (20.8%), triple negative (18.2%) and HER2 (8.4%). Lastly, patients with a VUS on *BRCA1* or *BRCA2* had a majority of tumors of Luminal A/B1 types (54.7%), followed by triple negative (19.3%), Luminal B2 and HER2 (13% each).

When the age at diagnosis was compared with the mutational status of *BRCA1/BRCA2, it* was observed that, among the group with germline mutations in *BRCA1*, the mean age at diagnosis was 42.1 years (SD = 11.1, CI = 23–77 years) and for the group with BRCA2 mutations, the mean age was 44.7 years (SD = 11.5, CI = 26–67 years) (Supplementary Table S1 and Supplementary Figure S1).

### Cancer family history

Among the 349 families evaluated, 85 (24.5%) reported the presence of at least one case of OC among first, second or third degree relatives (or in the proband itself), 26 (7.5%) had bilateral breast cancer (BBC), 11 families (3.2%) reported cases of male BC, and 18 families (5.2%) had history of pancreatic cancer.

We observed an average of three generations affected by cancer (SD = 5), and 5 cancer cases per family (SD = 6), with a mean of 3 BC cases (SD = 5). When considering mutated families, the average of BC among cases with *BRCA1* germline mutations was 2.8 (SD = 1.8), while for *BRCA2* mutated cases this mean was 3.4 (SD = 3.0).

There was a statistically significant difference in the distribution of BBC cases according to the status of *BRCA1/BRCA2* mutations. When the analysis was stratified by gene, it was observed that the presence of BBC was associated with the presence of germline mutations in *BRCA1* (*p* = 0.002), with 8/49 families with *BRCA1* mutation presenting BBC cases in the family.

### Mutational profile

**Figure 1:**
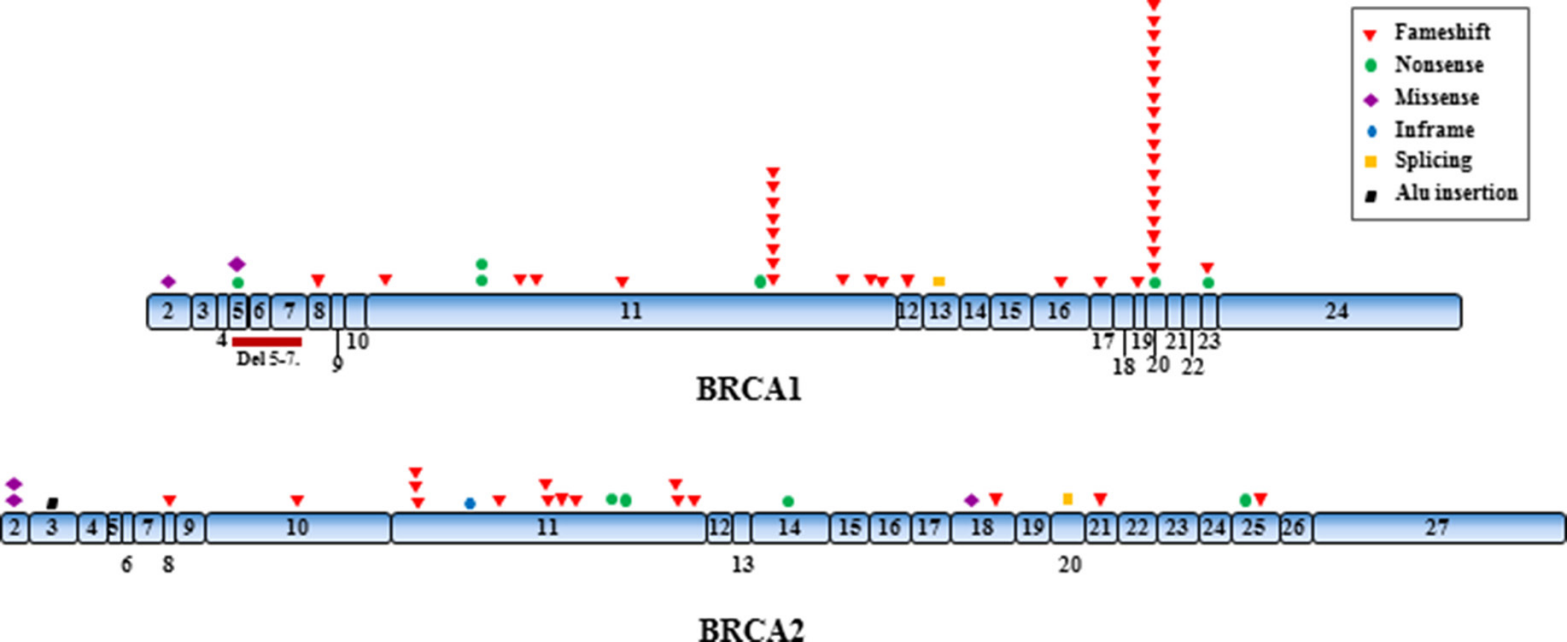
Distribution of the identified mutations along the *BRCA1* and *BRCA2* genes

Among the 349 index cases included in the study, 75 (21.5%) showed pathogenic germline mutations in *BRCA1* (*n* = 49) or *BRCA2* (*n* = 26) (Table 1). No patient carrying more than one pathogenic variant was identified. The distribution of the different types of mutations (missense, nonsense, frameshift, splicing and rearrangements) along the exons as well as its frequency are shown in Figure 1 and Supplementary Figure S2 respectively.

**Table 1:** Germline mutations identified (pathogenic)

Mutation	Mutated Gene	Number of families	Mutation Type	Tumors in index patient, age at onset^*^	Number of BC cases in relatives^*^	Number of OC cases in relatives^*^
c.65T>C p.Leu22Ser	*BRCA1*	1	Missense	Ovarian, 55	0	0
c.181T>G p.Cys61Gly	*BRCA1*	1	Missense	Breast, 59	5	1
c.188T>A p.Leu63Ter	*BRCA1*	1	Nonsense	Breast, 26	4	0
c.470_471delCT p.Ser157Ter	*BRCA1*	1	Missense	Breast, 43	5	0
c.1088delA p.Asn363Ilefs^*^11	*BRCA1*	1	Frameshift	Breast, 36	2	0
c.1687C>T p.Gln563Ter	*BRCA1*	2	Nonsense	Breast, 29; Breast, 48	4; 3	0; 0
c.1912delG "p.Glu638Asnfs^*^13"	*BRCA1*	1	Frameshift	Breast, 53	3	0
c.1962dupG p.Tyr655Valfs^*^18	*BRCA1*	1	Frameshift	Breast, 34	3	0
c.2405_2406delTG p.Val802Glufs^*^7	*BRCA1*	1	Frameshift	Breast, 35	2	0
c.3239T>A p.Leu1080Ter	*BRCA1*	1	Nonsense	Breast, 31	1	0
c.3331_3334delCAAG p.Q1111Nfs^*^5	*BRCA1*	8	Frameshift	Breast, 39; Ovarian, 32; Breast, 32; Breast, 46; Breast, 47; Breast, 30; Ovarian, 54; Ovarian, 53	2; 1; 4; 4; 3; 5; 0; 0	0; 0; 1; 0; 2; 0;0; 0
c.3764dupA p.Asn1255Lysfs^*^11	*BRCA1*	1	Frameshift	Breast, 35	3	0
c.3916_3917delTT p.Leu1306Aspfs^*^23	*BRCA1*	2	Frameshift	Ovarian, 52; breast, 32	3; 1	2; 0
c.4165_4166delAG p.Ser1389Ter	*BRCA1*	1	Frameshift	Breast, 33	2	0
c.4357+1G>C	*BRCA1*	1	Splicing	Breast, 47	3	0
c.4964_4982del19 p.Ser1655Tyrfs^*^16	*BRCA1*	1	Frameshift	Breast, 29	2	0
c.5030_5033delCTAA p.Thr1677Ilefs^*^2	*BRCA1*	1	Frameshift	Breast, 40	4	0
c.5161delC p.Gln1721Serfs^*^9	*BRCA1*	1	Frameshift	Breast, 45	1	1
c.5251C>T p.Arg1751Ter	*BRCA1*	1	Nonsense	Breast, 23	4	1
c.5266dupC p.Gln1756Profs^*^74	*BRCA1*	18	Frameshift	Breast, 36; Breast, 63; Ovarian, 77; Breast, 36; Breast, 44; Ovarian, 54; Ovarian, 47; Ovarian, 53: Breast, 31; Ovarian, 47; Breast, 41; Ovarian, 49; Breast, 33; Breast, 42; Breast, 37; Breast, 28; Breast, 28; Breast, 47	8; 4; 2; 2; 4; 2; 0; 4; 2; 0; 5; 0; 6; 3; 2; 4; 1; 4	0; 0; 0; 1; 0; 0; 0; 1; 0; 0; 0; 0; 1; 0; 0; 0; 0; 0
c.5444G>A p.Trp1815Ter	*BRCA1*	1	Nonsense	Breast, 45	6	0
c.5463_5464insT p.His1822Serfs^*^7	*BRCA1*	1	Frameshift	Breast, 50	4	1
deletion exons 5 to 7	*BRCA1*	1	Rearrangement	Breast, 56	2	1
c.2T>G p.Met1Arg	*BRCA2*	2	Missense	Breast, 48; Breast, 43	6; 5	0; 0
c.156_157insAlu	*BRCA2*	1	Rearrangement	Breast, 29	2	0
c.658_659delGT p.Val220Ilefs^*^4	*BRCA2*	1	Frameshift	Breast, 49	2	1
c.1138delA p.Ser380Valfs^*^19	*BRCA2*	1	Frameshift	Breast, 49	4	0
c.2808_2811delACAA p.Ala938Profs^*^21	*BRCA2*	3	Frameshift	Breast, 43; Breast, 34; Breast, 36	2; 4; 2	0; 0; 0
c.3858_3860delAAA p.Lys1286del	*BRCA2*	1	In frame deletion	Breast, 49	2	0
c.4284dupT p.Gln1429Serfs^*^9	*BRCA2*	1	Frameshift	Ovarian, 65	0	0
c.5073dupA p.Trp1692Metfs^*^3	*BRCA2*	2	Frameshift	Ovarian, 63; Breast, 31	1; 1	0; 1
c.5158_5159insA p.Ser1720Tyrfs^*^7	*BRCA2*	1	Frameshift	Breast, 51	1	1
**c.5216dupA p.Tyr1739Ter**	*BRCA2*	1	Frameshift	Bresat, 36	3	0
c.5682C>G p.Tyr1894Ter	*BRCA2*	1	Nonsense	Breast, 44	6	0
c.5857G>T p.Glu1953Ter	*BRCA2*	1	Nonsense	Ovarian, 56	3	1
c.6405_6409delCTTAA p.Asn2135Lysfs^*^2	*BRCA2*	2	Frameshift	Breast 41; Breast, 48	7; 4	0; 0
c.6611delC p.Pro2204Leufs^*^2	*BRCA2*	1	Frameshift	Breast, 46	3	0
c.7180A>T p.Arg2394Ter	*BRCA2*	1	Nonsense	Breast, 34	1	0
c.7987delG p.Glu2663Lysfs^*^10	*BRCA2*	1	Frameshift	Breast, 31	7	0
c.8023A>G p.Ile2675Val	*BRCA2*	1	Missense	Ovarian, 59	3	0
c.8488-1G>A	*BRCA2*	1	Splicing	Breast, 26	1	0
c.8711delT p.Leu2904Leufs^*^4	*BRCA2*	1	Frameshift	Breast, 30	4	1
c.9382C>T p.Arg3128Ter	*BRCA2*	1	Nonsense	Breast, 53	3	1
c.9401delG p.Gly3134Alafs^*^29	*BRCA2*	1	Frameshift	Breast, 35	5	0

Bold indicates new mutations identified in the present study.

* cases where more the one family carries the same mutation are separated by “;”.

Among the mutated patients, 60 had a personal history of BC and 15 of OC. About the BC with a *BRCA1* mutation, the great majority were invasive ductal carcinomas. For *BRCA2* mutated patients, 82% were invasive ductal carcinomas, 14% lobular invasive carcinomas and 4% were medular tumors. Regarding OC patients with a *BRCA1* mutation, 67% were high grade serous adenocarcinoma, and 33% were undifferentiated. For the *BRCA2* mutated, the predominant pathology were also the serous adenocarcinoma (50%), followed by one case of peritoneal tumor and one of undifferentiated ovarian tumor.

For *BRCA1,* 21 different pathogenic mutations were identified and one of those was present in 18 different families, representing 36.7% of the *BRCA1* mutated families: the founder mutation c.5266dupC, previously known as 5382insC, in exon 20 of *BRCA1* gene. The second most common mutation (shared by 8 families) was the frameshift alteration c.3331_3334delCAAG in exon 11 of *BRCA1*. These mutations were identified in families coming from different Brazilian regions: 64% from Southeast, 28% from Central-West and 8% came from the North region of the Brazilian territory.

Twenty-two distinct mutations were identified in *BRCA2.* The only change shared by more than two families was the frameshift mutation c.2808_2811delACAA in exon 11 of this gene, present in three families. Among the identified mutations, two were rearrangements: a deletion of exons 5 to 7 at *BRCA1* identified by MLPA analysis and an Alu insertion at *BRCA2* exon 3, identified by a mutation-specific PCR.

An association between the presence of c.5266dupC mutation and the occurrence of BBC was observed: 22.7% of families with this mutation had BBC, in the proband (16.7%) or in the family (11.1%) (OR: 3.987, 95% CI, 1.210–13.137, *p* = 0.016). Additionally, there was an association between the presence of this mutation and the development of OC (OR: 3.2, 95% CI, 1.1–9.0) (*p* = 0.023).

Six new mutations were identified (not described in the HGMD, BIC, UMD and ClinVar databases): 2 in *BRCA1* (c.1962dupG and c.5161delC) and four in *BRCA2* (c.5158_5159insA, c.5216dupA, c.7987delG and c.8711delT). All new mutations identified are frameshift alterations and lead to the formation of an altered and probably non-functional protein. Besides, 39 out of the 44 pathogenic mutations identified in our study (88.6%) were not previously reported in the Brazilian population.

Regarding missense variants, there was an extensive review of databases and *in silico* analysis, in order to classify those alterations according to its possible pathogenicity. As shown in Table 2, there is a large inconsistency among the databases considered for a large proportion of the variants analyzed. In addition, data regarding the frequency with which the change has been identified (number of families with the given alteration) and the co-segregation with proven pathogenic variants is reported. As detailed in Table 3, five variants were identified in patients carrying pathogenic mutations. The variants c.811G>A (*BRCA1*) and c.8187G>T (*BRCA2*) were identified in a patient carrying the pathogenic mutation c.8023A>G (p.Ile2675Val) at the *BRCA2* gene. In addition, the alteration c.8351G>A (*BRCA2*) was identified in two patients and one of them is carrier of the mutation p.Arg337His at *TP53* gene (this patient also has the variant c.7469T>C, at *BRCA2* gene). Lastly, the alteration c.7448G>A (BR*CA2)* was identified in a patient carrying the *BRCA1* mutation c.1687C>T(p.Gln563Ter).

**Table 2:** Variants of unknown significance according to 7 databases an 5 *in silico* prediction programs

	HGMD	BIC	LOVD	LOVD IARC	ClinVar	ARUP	BRCA Share™	Polyphen- 2	SIFT	Align- GVGD	MAPP	CADD
***BRCA1***												
c.190T>C; p.Cys64Arg	Yes	VUS	Normal RNA splicing	N/R	conflicting data	N/R	Causal	probably damaging	Predict Not Tolerated	C65	Not tolerated	NC
c.811G>A; p.Val271Met	Yes	VUS	probably neutral	N/R	VUS	N/R	N/R	possibly damaging	Predict Tolerated	C0	Not tolerated	NC
c.1601A>G; p.Gln534Arg	N/R	VUS	N/R	N/R	VUS	N/R	VUS	benign	Predict Not Tolerated	C0	Not tolerated	High risk
c.1648A>C p.Asn550His	Yes	VUS	probably neutral	class 1	conflicting data	Class 1	Neutral	probably damaging	Predict Not Tolerated	C0	Tolerated	NC
c.3823A>G p.Ile1275Val	N/R	VUS	probably neutral	N/R	conflicting data	N/R	Neutral	benign	Predict Tolerated	C0	Tolerated	NC
c.4484G>A p.Arg1495Lys	VUS	yes	N/R	N/R	conflicting data	N/R	Causal	benign	Predict Not Tolerated	C0	Not tolerated	NC
c.5062_5064delGTT; p.Val1688del	Yes	VUS	No splicing defect	N/R	conflicting data	Class 5	Causal	NC	NCR	NC	NC	NC
c.5096G>A p.Arg1699Gln	Yes	VUS	probably deleterious	N/R	conflicting data	N/R	VUS	probably damaging	Predict Not Tolerated	C35	Not tolerated	Medium risk
c.5153-2A>C	N/R	N/R	N/R	N/R	N/R	N/R	N/R	NC	NC	NC	NC	NC
c.5425_5430delGTTGTG	N/R	VUS	N/R	N/R	VUS	N/R	N/R	NC	NC	NC	NC	NC
c.5509T>C; p.Trp1837Arg	Yes	VUS	probably deleterious	N/R	conflicting data	N/R	VUS	probably damaging	Predict Not Tolerated	C65	Not tolerated	NC
***BRCA2***												
c.223G>C p.Ala75Pro	Yes	VUS	probably neutral	class 1	conflicting data	Class 1	Neutral	probably damaging	Predict Not Tolerated	C0	Not tolerated	NC
c.1798T>C p.Tyr600His	N/R	VUS	N/R	N/R	conflicting data	N/R	VUS	benign	Predict Tolerated	C0	Tolerated	NC
c.2274 T > G p.Ser758Arg	N/R	N/R	N/R	N/R	VUS	N/R	N/R	benign	Predict Tolerated	C0	Not tolerated	Low risk
c.2350A>G p.Met784His	VUS	VUS	probably neutral	class 3	N/R	Class 3	VUS	possibly damaging	Predict Not Tolerated	C0	Not tolerated	NC
c.2503C>T p.Pro835Ser	N/R	VUS	N/R	N/R	VUS	N/R	N/R	benign	Predict Tolerated	C0	Tolerated	Low risk
c.4585G>A p.Gly1529Arg	Yes	no	inconclusive	class 1	benign	Class 1	Neutral	probably damaging	Predict Not Tolerated	C65	Tolerated	NC
c.4681C>A p.His1561Asn	N/R	VUS	N/R	N/R	conflicting data	N/R	VUS	benign	Predict Tolerated	C0	Tolerated	NC
c.4928T>C p.Val1643Ala	N/R	VUS	inconclusive	class 3	conflicting data	Class 3	VUS	benign	Predict Tolerated	C0	Tolerated	NC
c.5096A>G p.Asp1699Gly	N/R	VUS	N/R	N/R	VUS	N/R	N/R	benign	Predict Tolerated	C0	Not tolerated	NC
c.5640T>G p.Asn1880Lys	Yes	VUS	probably neutral	N/R	conflicting data	N/R	Polymorphism	benign	Predict Not Tolerated	C0	Not tolerated	NC
c.6347A>G p.His2116Arg	Yes	no	N/R	N/R	benign	N/R	Neutral	possibly damaging	Predict Not Tolerated	C0	Tolerated	NC
c.6412G>T p.Val2138Phe	N/R	VUS	probably neutral	N/R	conflicting data	N/R	VUS	benign	Predict Not Tolerated	C0	Tolerated	NC
c.6554C>T p.Ala2185Val	N/R	N/R	N/R	N/R	N/R	N/R	N/R	benign	Predict Tolerated	C0	Tolerated	Low risk
c.6935A>T p.Asp2312Val	VUS	VUS	probably neutral	N/R	VUS	N/R	VUS	probably damaging	Predict Not Tolerated	C65	Not tolerated	NC
c.6988A>G p.Ile2330Val	N/R	N/R	N/R	N/R	N/R	N/R	N/R	benign	Predict Tolerated	C0	Not tolerated	NC
c.7017G>C p.Lys2339Asn	VUS	no	N/R	N/R	conflicting data	N/R	Likely neutral	benign	Predict Tolerated	C0	Not tolerated	NC
c.7448G>A p.Ser2483Asn	N/R	VUS	probably neutral	N/R	conflicting data	N/R	VUS	benign	Predict Tolerated	C0	Tolerated	Low risk
c.7469T>C p.Ile2490Thr	VUS	no	probably neutral	N/R	conflicting data	N/R	VUS	benign	Predict Tolerated	C45	Not tolerated	NC
c.7507G>A p.Val2503Ile	N/R	N/R	N/R	N/R	VUS	N/R	VUS	benign	Predict Tolerated	C0	Tolerated	Low risk

**Table 3:** Information regarding tumor history, frequency and segregation of the variants of unknown significance

	NOF	Co-segregation	Tumors in index patient, age at onset^*^	Number of BC cases in relatives^*^	Number of OC cases in relatives^*^
***BRCA1***					
c.190T>C; p.Cys64Arg	1	no	Breast, 30	2	0
c.811G>A; p.Val271Met	1	yes	Ovarian, 59	3	1
c.1601A>G; p.Gln534Arg	1	no	Breast, 51	5	0
c.1648A>C p.Asn550His	1	no	Breast, 49	4	0
c.3823A>G p.Ile1275Val	1	no	Breast, 43	1	0
c.4484G>A p.Arg1495Lys	1	no	Breast, 26	3	0
c.5062_5064delGTT; p.Val1688del	1	no	Breast, 33	2	0
c.5096G>A p.Arg1699Gln	1	no	Ovarian, 44	0	1
c.5153-2A>C	1	no	Breast, 28	1	0
c.5425_5430delGTTGTG	1	no	Breast, 36	7	0
c.5509T>C; p.Trp1837Arg	1	no	Breast, 28	0	0
***BRCA2***					
c.223G>C p.Ala75Pro	2	no	Breast, 20; Breast, 54	1;3	0;0
c.1798T>C p.Tyr600His	1	no	Ovarian, 46	0	0
c.2274 T > G p.Ser758Arg	1	no	Breast, 35	2	0
c.2350A>G p.Met784His	1	no	Breast, 55	3	0
c.2503C>T p.Pro835Ser	2	no	Breast, 54	2	0
c.4585G>A p.Gly1529Arg	1	no	Breast, 27	2	0
c.4681C>A p.His1561Asn	1	no	Breast, 28	1	0
c.4928T>C p.V1643A	1	no	Breast, 40	2	0
c.5096A>G p.Asp1699Gly	1	no	Breast, 24	1	0
c.5640T>G p.Asn1880Lys	2	no	Breast, 27; Breast, 36	1;2	0;0
c.6347A>G p.His2116Arg	3	no	Breast, 38; Breast, 26; Breast, 44	4;1;4	0;0;?
c.6412G>T p.Val2138Phe	1	no	Breast, 49	2	0
c.6554C>T p.Ala2185Val	1	no	Breast, 31	2	0
c.6935A>T p.Asp2312Val	1	no	Breast, 28	1	0
c.6988A>G p.Ile2330Val	1	no	Breast, 24	2	0
c.7017G>C p.Lys2339Asn	2	no	Ovarian, 33; Breast, 74	2;1	1;0
c.7448G>A p.Ser2483Asn	1	yes	Breast, 29	4	0
c.7469T>C p.Ile2490Thr	10	yes	Breast, 29; Breast, 39; Breast, 29; Breast, 38; Breast, 43; Breast, 40; Breast, 37; Breast, 41; Bresat, 61; Breast, 29;	1;3;1;1;4;2;6;1;3;1	0;0;0;0;0;0;0;0;0;0
c.7507G>A p.Val2503Ile	1	no	Breast, 37	2	0
c.7994A>G p.Asp2665Gly	1	no	Breast, 60	2	1
c.8187G>T P.Lys2729Asn	1	yes	Ovarian, 59	3	0
c.8351G>A p.Arg2784Gln	2	yes	Breast, 41; Breast, 40	1;2	0;0
c.8755G>T p.Gly2919Cys	1	no	Breast, 32	0	0
c.9235G>A p.Val3079Ile	1	no	Breast, 55	2	0
c.9730G>A p.Val3244Ile	2	no	Ovarian, 33; Breast, 74	2;1	1;0

* cases where more the one family carries the same mutation are separated by “;”.

Furthermore, three patients harbor more than one variant. One of them is carrier of the c.7469T>C (*BRCA2*) and c.8351G>A (*BRCA2*) variants (this patient also have the pathogenic mutation p.Arg337His at *TP53* gene as described above); the other have the alterations c.4928T>C (*BRCA2*), and c.8351G>A (*BRCA2*). In addition one patient (with OC at 59 years of age and carrier of the pathogenic mutation c.8023A>G (p.Ile2675Val) at the *BRCA2* gene) has two variants: c.811G>A (*BRCA1*) and c.8187G>T (*BRCA2*).

The identification of two families with possible pathogenic variants (C65 score) in *BRCA1* (c.190T>C, p.Cys64Arg, c.5509T>C and p.Trp1837Arg) as well as three families carrying variants with the same score in *BRCA2* (c.4585G>A, p.Gly1529Arg; c.6935A>T, p.Asp2312Val, c.7994A>G and p.Asp2665Gly) was possible using the AlignGVGD program. The probands of four out of these 5 families have had BC at very early ages (ages between 27 and 30 years as can be seen in details at Table 3). The only family with tumors at more advanced ages among those with variants classified as C65 is the family with the variant c.7994A>G at *BRCA2* gene, where the proband had BC at 60 years old. However, this woman has two relatives with BC (at 35 and 66 years respectively) and one with OC (age at diagnosis unknown).

An alteration located in a splicing region in the *BRCA1* gene (variant c.5153-2A>C) was also identified in a patient with BC at 28 years old. However, since there was no previous report in the literature, as well as no functional study performed supporting their non-functionality, this variant was not considered as proven pathogenic. Similarly, the *BRCA2* variants (missense) c.6554C>T (p.Ala2185Val), c.6988A>G (p.Ile2330Val) and c.8755G>T (p.Gly2919Cys) have not been previously described in the literature and given the lack of information about their biological impact they have been “provisionally” considered VUS. However it is important to highlight that the patient with the variant c.6988A>G (*BRCA2*) had a personal history of BBC at 24 years of age and 2 relatives with BC, raising strong suspicion about its pathogenicity (although classified as benign by the *in silico* tools used).

Two other identified variants that call our attention were: (i) the c.5096G>A (*BRCA1*), identified in a family without BC history (proband with OC at 44 and one relative with OC), which was the only variant identified in families with cancer history exclusive of OC and (ii) the *in frame* deletion c.5425_5430delGTTGTG, due to the severity of the family history (proband with BC at 36 years old and 7 cases of BC among first, second and third degree relatives). In the same way, two families with the alteration c.6347A>G (*BRCA2*) had probands with BC at 38 and 26 years respectively, and 4 cases of BC in each family were reported.

For the 274 index cases with no identified *BRCA1/BRCA2* germline mutation, test for the *TP53* gene was performed, which detected pathogenic mutation in 9 patients (data not shown). All these 9 families had probands with BC diagnosed before the age of 50 (varying from 27 to 48 years) and at least one first or second degree relative with BC. All 9 families are carriers of the Brazilian founder mutation p.Arg337His (exon 10 of *TP53* gene) [21].

### Ancestry

**Figure 2:**
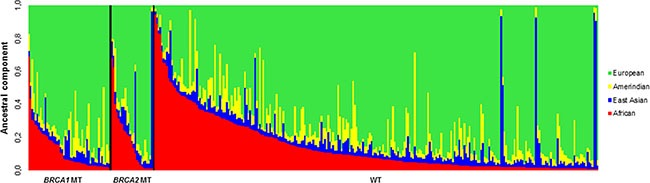
Ancestral profile of the studied patient group showing individual ancestry estimates according to mutational status *BRCA1* mutated (left), *BRCA2* mutated (centre) and *BRCA1/BRCA2* WT (right).

We further performed genetic ancestry assessment on 341 of the 349 participants (Figure 2) using 46 ancestry-informative markers. Although overall ancestry estimates computed with Admixture and Structure algorithms were similar, for simplicity we will refer only to the former one. Average ancestry proportions in our group were 70.6% European, 14.5% African, 8.0% Native American and 6.8% East Asian.

There was no statistically significant difference in the ancestry profile of WT individuals when compared to those with germline mutation in *BRCA1/BRCA2* genes (Supplementary Table S2 and Figure 2). Additionally, there is no difference in the ancestry profile between *BRCA1* and *BRCA2* mutated women (*p* = 0.434).

To verify the association of ancestry with clinical characteristics, patients were subdivided according to the *BRCA1/BRCA2* status: WT or mutated. Among the mutated ones (*BRCA1* or *BRCA2*), no association with clinico-pathological characteristics and genetic ancestry was observed.

On the other hand, we observed that the African ancestry component among individuals without *BRCA1/BRCA2* mutations was associated with higher tumor grade (*p* = 0.008). Furthermore, we found out that when the African component increases in an individual, the probability of BBC decreases (*p* = 0.005) and the number of cancer cases in the family increases (*p* = 0.04).

**Figure 3:**
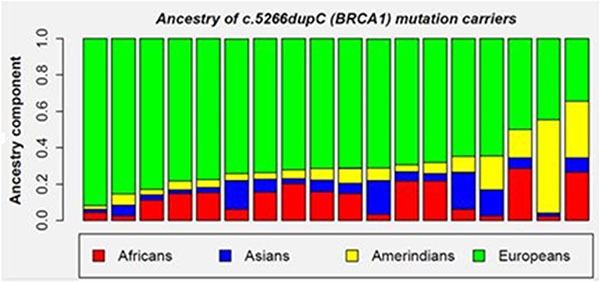
Ancestry contribute on of c.5266dupC mutated patients

In 15 out of 18 patients with c.5266dupC mutation in the *BRCA1* gene, the European ancestry component was predominant (over 69%) (Figure 3). There was no statistically significant difference in the ancestry component of patients carrying the c.5266dupC mutation vs. those with other *BRCA1/BRCA2* mutations. For one patient, there was a similar balance between the Amerindian (0.482) and European (0.478) components (Figure 3). In another family, there was a similar distribution among three components: 0.387 for the European, 0.280 for the Amerindian and 0.261 for the African component (Figure 3).

## DISCUSSION

The present work is the largest study analyzing the *BRCA1/BRCA2* germline mutation frequencies in the Brazilian population, with a prevalence of 21.5% of deleterious mutations, being 13.6% new mutations (not described in the literature) and 88.6% of them never described in the Brazilian population. Although it represents the data from a single Institution, the BCH receives patients from all over Brazil. In this particular study, the majority (71.6%) came from the Southeast region of Brazil, followed by the Central-West (18.3%), North (7.2%), and the Northeast and South regions (1.4% each), covering 16 Brazilian States. Moreover, given the highly admixed Brazilian population, and the potential impact of the presence of founder mutations, this study presents a characterization of the genetic ancestry of this cohort.

We observed a frequency of 21.5% *BRCA1/BRCA2* mutated patients, with most of the mutations located in the *BRCA1* gene (65.3%). Other than the results reported in this manuscript there are just a few studies on the subject carried out in the Brazilian population [7–13, 22–24] and they are depicted in Table 4.

**Table 4:** Summary of Brazilian *BRCA1* and/or *BRCA2* studies

Sample size	% of *BRCA1* /*BRCA*2 mutations	Selection criteria	Genetic test performed	Authors, year
47	7 (14.9%)	HBOC patients	All coding regions of *BRCA1*	Lourenço, 2004
31	4 (9.6%)	HBOC patients	*BRCA1* exons 2, 3, 5, 11 and 20 and *BRCA2* exons 10 and 11	Dufloth, 2005
402	9 (2.3%)	BC, irrespective to age or family history	Four common alterations in BRCA1 and BRCA2 (185delAG, 5382insC and exon 13 6kb duplication in BRCA1 and 6174delT in BRCA2	Gomes, 2007
612	21 (3.4%)	HBOC patients	BRCA1 exon 11 and BRCA2 exons 10 and 11 and specific founder mutations	Esteves, 2009
137	7 (5%)	non-Ashkenazyi HBOC patients	*BRCA1* exon 2 (c.68_69del mutation), exon 20 (c.5266dup mutation) and *BRCA2* exon 11 (c.5946del mutation).	Ewald, 2011
255	3 (1.2%)	Askenazi-jews unselected for family history of cancer	*BRCA1* 185delAG, *BRCA1* 5382insC and *BRCA2* 6174delT	Dillenburg, 2012
54	11 (20.4%)	BC <35years, irrespective to family history	All coding regions of *BRCA1* and *BRCA2*	Carraro, 2013
106	9 (8.5%)	HBOC patients	All coding regions of BRCA1 and common founder mutations in the BRCA2	Felix, 2014
120	27 (22.5%)	HBOC patients	*BRCA1/BRCA2* point mutations and rearrangements	Silva, 2014
616	0 (0%)	513 cancer-free patients and 103 OC patients	*BRCA1* (c.68_69delAG, c.5266dupC, c.181T>G, c.4034delA, c.5123C>A) and *BRCA2* (c.5946delT, c.8537_8538delAG, 4936_4939delGAAA, c.156_157insAlu)	Schayek,2015
**349**	**75 (21.5%)**	**HBOC patients**	**All coding regions of *BRCA1* and *BRCA2***	**Present study**

As shown in Table 4, there are only three studies involving the analysis of the entire coding region of the *BRCA1/BRCA2*: the study published by Carraro and colleagues in 2013 [24] that examined 54 women with BC diagnosed before the age of 35, the study published by Silva et al. in 2014 [23] which included 120 women and ours, that analyzed 349 index cases of suspected HBOC families. Although the inclusion criteria may differ, the three studies identified a similar percentage of mutations, 20.4%, 22.5% and 21.5% respectively. Other studies involving larger sample sizes were performed, however they investigated the presence of specific regions of the *BRCA1/BRCA2* genes or specific mutations (founder mutations), and the percentage of mutations identified were quite low [8, 11].

The fraction of pathogenic mutations identified in our study is in accordance with recent published studies all over the world. A study conducted by Peixoto et al. analyzed the entire *BRCA1*/*BRCA2* coding region of 524 Portuguese patients and identified 112/524 families (21.4%) with pathogenic mutation [25]. Kang and collaborators analyzed 2403 Korean index patients and found that the prevalence of *BRCA* mutations in BC patients with a family history of breast/ovarian cancer was 22.3% [26]. Churpek and colleagues [27] studied a group of 289 African-American women with family history of BC and the rate of identified pathogenic mutations was 22%. In a similar way, Weitzel and colleagues [28] evaluated 746 Hispanics with personal and/or family history of breast or OC, and found the presence of *BRCA1/BRCA2* mutations in 25% of them. In Colombia, a study conducted by Torres and collaborators found a frequency of 24.5% of deleterious *BRCA1*/*BRCA2* mutations in a cohort of 53 breast/ovarian cancer families evaluated [29]. Finally, data on 10 years of genetic testing (November 1996 to March 2006) conducted by Myriad Laboratory (USA), representing more than 95% of genetic testing for *BRCA1/BRCA2* genes performed in the United States, not-selected by breast/ovarian cancer family history, points to a frequency of 12.5% identified deleterious mutations (5,780 mutated individuals in a total of 46.276 individuals consecutively tested) [30].

In the present study we found that, among patients with mutated *BRCA1*, 77.5% had a personal history of BC, and 22.4% of OC. Regarding *BRCA2,* 84.6% had previous personal history of BC and 15.4% of OC. This is similar to the results recently published by Rebbeck and colleagues [31]. Rebbeck's results show that the proportion of cancer-affected individuals carrying mutated *BRCA1* with BC was 72.9%, with OC 18.7% and with a personal history of breast and ovarian cancer was 8.4%. Regarding *BRCA2*, 86.6% of the mutated patients had BC, 9.6% OC and 3.8% presented previous personal history of both tumors.

We observed that two *BRCA1* mutations are responsible for more than 50% of the mutations identified in this gene in our cohort: the c.5266dupC accounting for 36.7% of mutations in the *BRCA1* gene and for 24% of all mutations found in this study and the mutation c.3331_3334delCAAG, that represents 16.3% of our *BRCA1* mutations and 10.7% of the mutations identified. Although the present study is not representative of the Brazilian population diversity, the fact that i) two mutations account for over 50% of the identified mutations and ii) these two alterations were not concentrated in only one Brazilian region, may serve as a first approach strategy for genetic testing, not only in Brazil, but also in countries/services where the financial resources are limited and where the public health system or private plans do not cover such molecular analysis. If negative results are found with this initial screening, the analysis of all coding sequence of both *BRCA1* and *BRCA2* is imperative.

The presence of c.5266dupC mutation was associated with an increased risk for OC in our series, leading to a three times higher risk than those WT individuals for that mutation. Similarly, a study conducted in Poland identified that this mutation was present in 57% of the mutated OC cases [32].

The c.5266dupC mutation represents about 98–99% of the mutations found in Ashkenazi Jews, however it is not restricted to these populations. It has been reported as having high prevalence in several countries, mainly from Central and Eastern Europe [33]. Among the 18 patients in our study who are carriers of this mutation, although we could not assess the local ancestry of the locus containing the *BRCA1* c.5266dupC mutation, the European ancestry profile was prevalent in 94.4% of cases (17/18 families). For 83.3%, the European contribution was higher than 69%. In one case, the main contribution was the Amerindian followed by the European, demonstrating, once again, the wide miscegenation present in the Brazilian population.

Regarding genomic rearrangements, it is known that its frequency varies considerably among populations [34]. Although common in some populations, representing about 30% of the mutations identified in *BRCA1/BRCA2* genes in the Netherlands and Germany [35], large rearrangements are relatively rare in most populations [34]. In the present cohort, although not representative of the entire Brazilian population, only two families with rearrangements were identified, one case with rearrangement in *BRCA1* (deletion of exons 5–7), and the other with a rearrangement in *BRCA2* gene, that is regarded as a Portuguese founder mutation (c.156_157insAlu in *BRCA2*), mutation that represents about 40% of deleterious germline mutations of *BRCA1/BRCA2* in Portugal [33, 36, 37].

Concerning the variants with unknown clinical significance, we have reported a list of variants identified in our study (mostly missense variants), and, we observe a lack of consensus about their biological/clinical significance among the different existing databases. The number of cases tested in our center so far does not allow us to classify these variants based on their frequency or the co-occurrence of pathogenic mutations (although we have identified that five of our identified variants were present in individuals carrying pathogenic mutations, albeit not in the same gene for 4 of them) . Therefore, our strategy consists in *in silico* analysis, segregation studies (which will be conducted in cases where there is availability of family members with and without cancer) and analysis of available databases. However, the analysis of databases has not been instructive in most of cases. As an example, the *BRCA1* variants c.5509T>C and c.5096G>A have been considered pathogenic by HGMD, VUS, by BIC and UMD, “probably deleterious” by LOVD, and not conclusive by ClinVar (“conflicting data”). In the case of *BRCA2* variants c.4585G>A and c.6347A>G, these are considered to be pathogenic by HGMD, without clinical significance by BIC and benign by ClinVar. These discrepancies between the databases, although “explicable”, given the low prevalence of most of these variants, highly hinder the clinical work and the subsequent management of patients and their families. According to Miller-Samuel and colleagues “The VUS can create additional confusion and anxiety for the patient and family, and increased uncertainty for the clinician responsible for making medical management recommendations. Although clinicians are advised not to make management decisions based on VUS results, what actually happens in real-world clinical practice?” [38].

Even though clinician's decisions cannot be made based on VUS, some of our findings called our attention and deserve deep investigation. This is the case, for example for the age at diagnosis and the number of BC seen in the families carrying the alterations c.5425_5430delGTTGTG (*BRCA1*), c.6347A>G (*BRCA2*) and c.1601A>G (*BRCA1*) (8, 5 and 6 BC cases respectively), as well as the very early ages at diagnosis (24 years old) of the probands with the variants c.5096A>G (*BRCA2*) and c.6988A>G (*BRCA2*). Given the absence of consensus among databases, those families should be further evaluated through segregation analysis and functional studies. In addition, sequencing using other breast/ovarian cancer predisposition genes (high and moderate risk already present in commercial panels) should also be conducted.

It is also worth observing that the characteristic widespread miscegenation of the Brazilian population was proven in our sample. Although most of the cases analyzed present a major European ancestry component, we identified in all cases a contribution from more than one ancestor component, often equally distributed. Although study participants were from different regions of Brazil, we did not identify in this study a difference in the ancestry profile associated with the region of origin of the participants, which can be due to the high concentration of patients from the Southeast Brazilian region. According to Kedhy and colleagues [19], the African component in individuals of the northeast region of Brazil is of 50%, while in the South and Southeast of Brazil, the European component exceeds 70%. Still according to Kedhy and collaborators, in highly miscegenated Latin American populations such as the Brazilian, the frequency and distribution of deleterious mutations are determined more by the history of its population than for its demographics. In Brazil, for example, in 1870, Africans constituted the predominant ethnic group, which changed with the arrival of about 4 million Europeans during the second half of the 19th century and the first half of the 20th century. These migrations transformed Brazil in a predominantly white nation, especially in the South and Southeast regions of the country [19]. Nonetheless, although we found a great predominance of European ancestry in our sample, all individuals (mutated or not) had a greater or lesser extent of ancestral profiles admixture and this mixture was not correlated with mutational status, mutation type, age at diagnosis or type of cancer developed. However, some associations or trends could be observed, such as a more aggressive cancer behavior (higher grade tumor) in patients whose African component was larger. However, these data needs to be further validated in a larger sample group.

This study has some limitations, such as the fact that other high and moderated BC associated genes were not included in the test. In addition, although this is the largest Brazilian study involving complete sequencing of the genes *BRCA1/BRCA2* in a high-risk sample for HBOC and correlating the findings with the ancestral profile of the population., the cohort analyzed cannot be considered representative of the whole Brazilian population and the results obtained regarding the lack of association among the mutational profile and genetic ancestry should be further validated in a bigger cohort. The genotyping of a larger sample group with all five Brazilian regions equally represented would be very informative.

In conclusion, this is the largest Brazilian study involving complete sequencing of the genes *BRCA1/BRCA2* in a high-risk sample for HBOC and correlating the findings with the ancestral profile of the population. We identified 21.5% of *BRCA1/BRCA2* mutated patients, with most of the identified mutations located in the *BRCA1* gene (65.3%). Among the mutations identified, the mutations c.5266dupC and c.3331_3334delCAAG constitutes about 35% of the mutations identified in our cohort and more than 50% of the *BRCA1* mutations. In addition, we could not identify any association between the ancestry profile and the distribution of *BRCA1/BRCA2* mutations in the analyzed sample. Although genetic testing for these genes are already available for almost two decades, this test remains a great example of the use of human genetic variability in reducing the risk of developing cancer.

## MATERIALS AND METHODS

### Sample population

A total of 349 unrelated individuals at-risk for HBOC (with personal and familial history of BC and/or OC) were referred from the Oncogenetics Department (OD) for genetic testing for *BRCA1/BRCA2* genes in the period between 2010 and 2014. The OD was established in 2010 at the Barretos Cancer Hospital (reference center in Brazil for the prevention and treatment of cancer) and offers multidisciplinary care and in multiple steps, ranging from genetic risk assessment to genetic testing for high-risk families identified (Palmero EI, “in press”). All families were identified by the OD and fulfilled at least one of the following criteria: a) the clinical criteria recommended by the National Institute for Health Care Excellence (NICE) [39] (for details see Supplementary Table S3); b) probability of mutation in *BRCA1*/*BRCA2* over 20% according to the Myriad prevalence tables [40, 41]; c) BC diagnosed below 30 years old; and d) the presence of OC in the family. Furthermore, in the families where a pathogenic germline mutation was identified, mutation-specific predictive genetic test was performed for all interested relatives. Confirmation of the cancer family history was attempted in all cases by pathology and medical reports and/or death certificates. The project was approved by the local Ethics in Research Committee.

### *BRCA1* and *BRCA2* mutation screening

Genomic DNA was isolated from peripheral blood samples using the *QIAamp DNA Blood Mini Kit* (*Qiagen*) following the manufacturer's instructions. All coding exons were amplified by multiplex-PCR reactions, as described by Costa et al. [42].

After PCR amplification of the *BRCA1* (NM_007294.3) and *BRCA2* (NM_00059.3) genes, the products were purified (ExoSap-IT - *USB*), and sequenced (BigDye terminator v3.1 - *Applied Biosystems*). Following purification, samples were analyzed in a 3500xL Genetic Analyzer (*Applied Biosystems*). The results were analyzed using the SeqScape software (*Applied Biosystems*).

In addition, for samples analyzed after 2013, the sequencing of *BRCA1*/*BRCA2* was performed using the Ion Torrent PGM platform. For this, libraries containing the PCR product of 14 multiplex PCRs were sequenced using the Ion 316 chips, which allow the simultaneous analysis of 10 to 12 patients per chip. Data analysis was performed using *DNAstar Lasergene 10 software*, following parameters published by Costa et al. [42] and subsequently adapted by our group to the following: (1) Q call > = 40; (2) depth of coverage > = 100 and, (3) SNP % > = 23. All the identified variants were confirmed in a new PCR reaction followed by conventional bi-directional sequencing (Sanger).

The analysis of *BRCA1* and *BRCA2* rearrangements was performed with the Multiplex Ligation-dependent Probe Amplification Kit (MLPA) according to the manufacturer's protocol. For the presence of the c.156_157ins Alu at *BRCA2* gene, a mutation-specific PCR was performed following protocol described elsewhere [37].

### Mutations nomenclature and classification

The nomenclature of the identified sequence variants was applied following the guidelines of the Human Genome Variation Society (HGVS) [43]. The biological significance of the variants were verified in databases such as Human Genome Mutation Database (HGMD) [44], Breast Cancer Information Core (BIC) [45], LOVD IARC [46], CLINVAR [47], Universal Mutation Database (UMD, actual BRCA Share) [48], LOVD [49] and ARUP [50]. Only mutations clearly pathogenic or previously classified as such, were included. The Align-GVGD [51], Polyphen-2 [52], SIFT [53], MAPP [54] and CADD [55] algorithms were used for the *in silico* analysis of the variants of unknown clinical significance identified. All variants classified as VUS or with Conflicting results by Clinvar were considered as VUSs. In addition, variants with severe disagreement (classified as pathogenic by at least one database and benign or likely benign by others) were also included. All new variants (missense, in frame and splicing), not yet reported in the other databases consulted, were also considered VUSs.

### Ancestry analysis

In order to assess the genetic ancestry we employed a panel of 46 ancestry-informative markers (AIMs) selected to efficiently measure population admixture proportions of four different continental origins (African, European, East Asian and Native American) and that proved useful for the estimation of ancestral proportions in highly admixed individuals or populations like the Brazilian [56, 57]. The 46 AIMs were simply genotyped in one multiplex PCR followed by capillary electrophoresis as previously described [56]. Making use of published data for 748 individuals from the four continental ancestral references [56] plus 214 Brazilian control individuals previously characterized by our group [58] we then performed a comprehensive ancestry analysis in 341/349 patients. The clustering software Structure [59] was used to run supervised analyses using prior information on the origin of reference samples in order to estimate the ancestral components of the querying samples. Brazil is commonly considered as an essentially tri-hybrid (Native American, European, African) admixed population [19, 56, 57, 60–63], but there are particular locations in the country that harbor significant East Asian communities introduced more recently (e.g. São Paulo, Campinas; IBGE – Instituto Brasileiro de Geografia e Estatística, www.ibge.gov.br). For this reason, and taking into consideration the confirmed presence of individuals with East Asian ancestry among our patients, we proceeded with a conservative approach considering four possible ancestral contributors in our dataset (i.e. K = 4). Finally, we have performed equivalent analyses with Admixture [64] so as to assess the consistency of the ancestry estimates obtained with both programs.

### Statistical analysis

Statistical analysis was conducted using SPSS v.19.0 software (Chicago, IL). The comparisons between clinical, molecular and ancestral characteristics employed a simple analyses using the Chi-square (or Fisher's exact) test. Differences in ancestry estimates between mutated and non-mutated patients were carried out using Wilcoxon rank-sum test. The association of ancestry with patient characteristics (clinical and histopathological data and family history information) were examined with Mann-Withney (or Kruskal-Wallis) tests. The level of significance considered in all tests was 5%.

## SUPPLEMENTARY MATERIALS


