# Supplementary material for: Prevalence of BRCA1/BRCA2 mutations in a Brazilian population sample at-risk for hereditary breast cancer and characterization of its genetic ancestry
Source: Oncotarget. 2016 Oct 12;7(49):80465–81. doi: 10.18632/oncotarget.12610 (PMC5348334; doi:10.18632/oncotarget.12610)
Supplement: Supplementary file 1 [file oncotarget-07-80465-s001.pdf]

Prevalence of *BRCA1/BRCA2* mutations in a Brazilian population sample at-risk for hereditary breast cancer and characterization of its genetic ancestry

Supplementary Materials

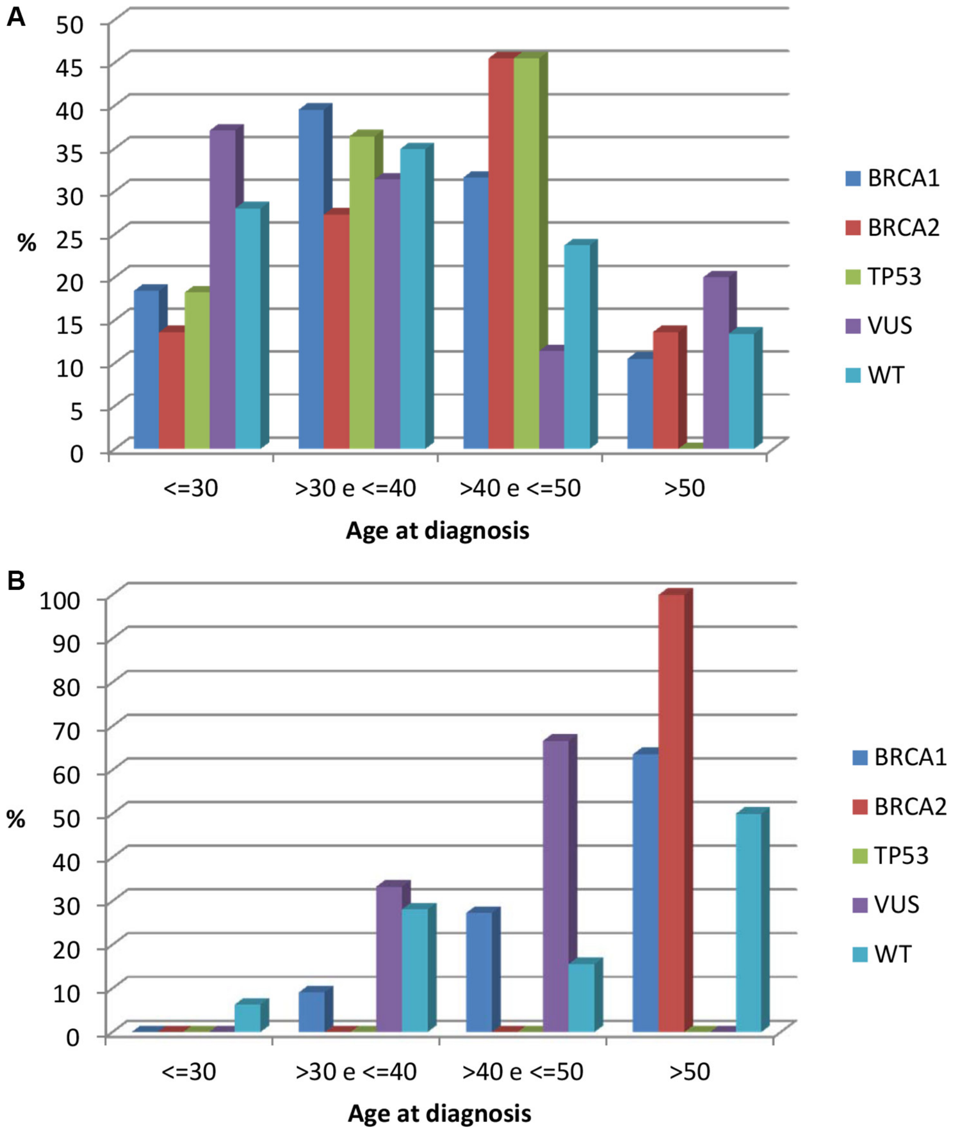

Supplementary Figure S1: Age at cancer diagnosis according to *BRCA1*, *BRCA2* and *TP53* status and cancer type. In (**A**) for breast cancer patients and, in (**B**), for ovarian cancer patients.

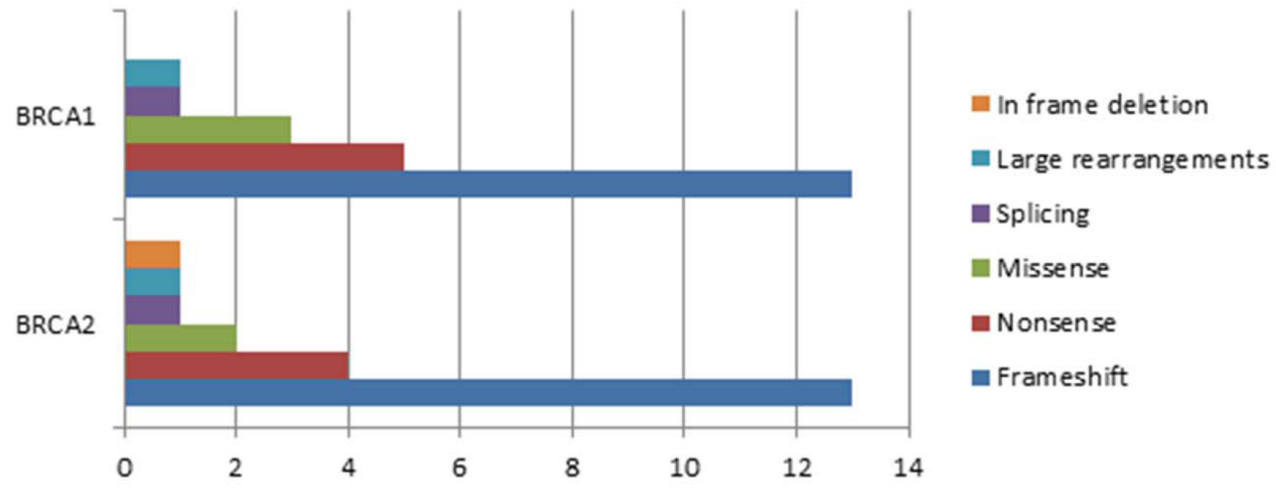

Supplementary Figure S2: Frequency of the different types of *BRCA1* and *BRCA2* mutations.

Supplementary Table S1: Clinical and histopathological features of the 349 patients

		mutation status						
		**All** *N* **(%)**	***BRCA1*** *N* **(%)**	***BRCA2*** *N* **(%)**	***TP53*** *N* **(%)**	**VUS** *N* **(%)**	**WT** *N* **(%)**	***p* value****
**Age at diagnosis breast cancer**	< = 30	77 (26.4)	7 (18.4)	3 (13.6)	1 (11.2)	13 (37.1)	52 (28)	0.172
	> 30 e < = 40	101 (34.6)	15 (39.5)	6 (27.3)	4 (44.4)	11 (31.4)	65 (34.9)	
	> 40 e < = 50	75 (25.7)	12 (31.6)	10 (45.5)	4 (44.4)	4 (11.4)	44 (23.7)	
	> 50	39 (13.3)	4 (10.5)	3 (13.6)	0 (0)	7 (20)	25 (13.4)	
**Age at diagnosis ovarian cancer**	< = 30	2 (4)	0 (0)	0 (0)	0 (0)	0 (0)	2 (6.3)	0.235
	> 30 e < = 40	11 (22)	1 (9.1)	0 (0)	0 (0)	1 (33.3)	9 (28.1)	
	> 40 e < = 50	10 (20)	3 (27.3)	0 (0)	0 (0)	2 (66.7)	5 (15.6)	
	> 50	27 (54)	7 (63.6)	4 (100)	0 (0)	0 (0)	16 (50)	
**Site of primary tumor**	Breast	292 (83.7)	38 (77.6)	22 (84.6)	9 (100)	35 (92.1)	186 (82.7)	0.452
	Ovarian	50 (14,3)	11 (22.4)	4 (15.4)	0 (0)	3 (7.9)	32 (14.2)	
	Other tumor	7 (2,0)	0 (0)	0 (0)	0 (0)	0 (0)	7 (3.1)	
**Histological grade***	grade I	27 (10.4)	1 (3.1)	2 (10.5)	0 (0)	3 (10)	21 (12.3)	0.555
	grade II	121 (46.5)	12 (37.5)	11 (57.9)	4 (50)	16 (53.3)	78 (45.6)	
	grade III	112 (43.1)	19 (59.4)	6 (31.6)	4 (50)	11 (3.7)	72 (42.1)	
**cTNM classification**	Tis	27 (9.1)	4 (9.8)	4 (18.2)	1 (11.1)	6 (16.7)	12 (6.3)	0.741
	T1	89 (30)	14 (34.1)	4 (18.2)	4 (44.4)	9 (25)	58 (30.7)	
	T2	77 (25.9)	10 (24.4)	8 (36.4)	1 (11.1)	10 (27.8)	48 (25.4)	
	T3	61 (20.5)	6 (14.6)	4 (18.2)	2 (22.3)	7 (19.4)	42 (22.2)	
	T4	43 (14.5)	7 (17.1)	2 (9)	1 (11.1)	4 (11.1)	29 (15.3)	
**cTNM classification**	N0	167 (55.1)	22 (52.4)	14 (60.9)	3 (33.3)	18 (50)	110(57)	0.598
	N 1 2 3	136 (44.9)	20 (47.6)	9 (39.1)	6 (66.7)	18 (50)	83 (43)	
**cTNM classification**	M0	275 (92)	36 (90)	21 (87.5)	8 (88.9)	32 (97)	178 (92.2)	0.592
	M1	24 (8)	4 (10)	3 (12.5)	1 (11.1)	1 (3)	15 (7.8)	
**Gender**	Female	340 (97.4)	49 (100)	26 (100)	9 (100)	36 (94.7)	218 (96.9)	0.559
	Male	9 (2.6)	0 (0)	0 (0)	0 (0)	2 (5,3)	7 (3,1)	

*For breast cancer only; **chi-squared test or Fisher´s exact test.

Supplementary Table S2: Genetic ancestry according to the *BRCA1/BRCA2* mutational status

Ancestry	WT			VUS			*BRCA1* mutated			*BRCA2* mutated		
	Median	Media	CI	Median	Media	CI	Median	Media	CI	Median	Media	CI
**African**	0.083	0.1467	0.119–0.164	0.115	0.178	0.119–0.237	0.066	0.1239	0.087–0.160	0.182	0.189	0.112-0.265
**European**	0.783	0.711	0680–0.741	0.751	0.642	0.562–0721	0.747	0.725	0.678–0.772	0.664	0.638	0.530-0.747
**Asiatic**	0.038	0.067	0.052–0.082	0.042	0.089	0.037–0.142	0.044	0.056	0.043–0.069	0.056	0.112	0.030-0.194
**Amerindian**	0.047	0.079	0.067–0.092	0.046	0.089	0.065–0.113	0.053	0.094	0.065–0.122	0.046	0.059	0.039-0.080

Supplementary Table S3: NICE guidelines (CG164) for familial breast cancer

At least the following female breast cancers only in the family:

- two first-degree or second-degree relatives diagnosed with breast cancer at younger than an average age of 50 years (at least one must be a first-degree relative) or
- three first-degree or second-degree relatives diagnosed with breast cancer at younger than an average age of 60 years (at least one must be a first-degree relative) or
- four relatives diagnosed with breast cancer at any age (at least one must be a first-degree relative). or

Families containing one relative with ovarian cancer at any age and, on the same side of the family:

- one first-degree relative (including the relative with ovarian cancer) or second-degree relative diagnosed with breast cancer at younger than age 50 years or
- two first-degree or second-degree relatives diagnosed with breast cancer at younger than an average age of 60 years or
- another ovarian cancer at any age. or

Families affected by bilateral cancer (each breast cancer has the same count value as one relative):

- one first-degree relative with cancer diagnosed in both breasts at younger than an average age 50 years or
- one first-degree or second-degree relative diagnosed with bilateral cancer and one first or second degree relative diagnosed with breast cancer at younger than an average age of 60 years. or

Families containing male breast cancer at any age and, on the same side of the family, at least:

- one first-degree or second-degree relative diagnosed with breast cancer at younger than age 50 years or
- two first-degree or second-degree relatives diagnosed with breast cancer at younger than an average age of 60 years. or

A formal risk assessment has given risk estimates of:

- a 10% or greater chance of a gene mutation being harboured in the family or
- a greater than 8% risk of developing breast cancer in the next 10 years or
- a 30% or greater lifetime risk of developing breast cancer.
